# Toward a Theory of the Evolution of Fair Play

**DOI:** 10.3389/fpsyg.2018.01167

**Published:** 2018-07-24

**Authors:** Jeffrey C. Schank, Gordon M. Burghardt, Sergio M. Pellis

**Affiliations:** ^1^Department of Psychology, University of California, Davis, Davis, CA, United States; ^2^Departments of Psychology and Ecology & Evolutionary Biology, University of Tennessee, Knoxville, Knoxville, TN, United States; ^3^Department of Neuroscience, University of Lethbridge, Lethbridge, AB, Canada

**Keywords:** social play, fairness, cooperation, evolutionary game theory, equitability, social development

## Abstract

Juvenile animals of many species engage in social play, but its functional significance is not well understood. This is especially true for a type of social play called fair play (Fp). Social play often involves behavioral patterns similar to adult behaviors (e.g., fighting, mating, and predatory activities), but young animals often engage in Fp behaviors such as role-reversals and self-handicapping, which raises the evolutionary problem of why Fp exists. A long-held working hypothesis, tracing back to the 19th century, is that social play provides contexts in which adult social skills needed for adulthood can be learned or, at least, refined. On this hypothesis, Fp may have evolved for adults to acquire skills for behaving fairly in the sense of equitable distribution of resources or treatment of others. We investigated the evolution of Fp using an evolutionary agent-based model of populations of social agents that learn adult fair behavior (Fb) by engaging in Fp as juveniles. In our model, adults produce offspring by accumulating resources over time through foraging. Adults can either behave selfishly by keeping the resources they forage or they can pool them, subsequently dividing the pooled resources after each round of foraging. We found that fairness as equitability was beneficial especially when resources were large but difficult to obtain and led to the evolution of Fp. We conclude by discussing the implications of this model, for developing more rigorous theory on the evolution of social play, and future directions for theory development by modeling the evolution of play.

## Introduction

Many species of animals engage in social play as juveniles and even in adulthood (Fagen, 1981; Palagi, 2011), but its functional significance is not well understood and accounting for its evolution has proven challenging (Caro, 1988; Burghardt, 2005). Social play appears not to be adaptive, especially in immature animals, because typically no immediate functions are apparent (Martin and Caro, 1985), it is costly due to increased mortality from predation, injury, and disease (e.g., Harcourt, 1991; Kuehl et al., 2008). However, it can have immediate benefits in terms of exercise, metabolism, and perceptual-motor coordination among other possibilities (see Burghardt, 2005 for review). A common working hypothesis, going back to the instinct-practice views of Groos (1898), is that there must be more adaptive benefits to social play and that these benefits come from learning specific social skills as juveniles that will be useful during adulthood (Pellis and Pellis, 2009; Pellis et al., 2010). While there is some limited empirical evidence supporting this working hypothesis (Pellis et al., 2014; Vanderschuren and Trezza, 2014), until recently, there has been little theoretical support for it. Durand and Schank (2015), using an evolutionary agent-based modeling approach, showed that learning to cooperate by engaging in social play could stably evolve even with relatively high costs of mortality. Their model demonstrated that the synergistic benefits of learning to cooperate as adults via social play as juveniles can outweigh the costs of social play.

That the synergistic benefits of adult cooperation can outweigh the costs of social play is intuitively clear, but a more controversial idea is that young animals engaging in fair play (Fp) acquire skills needed to behave fairly as adults (Bekoff, 2001; Palagi et al., 2016). Bekoff (2001) has argued that behaviors such as self-handicapping (e.g., an individual not biting as hard as it can) and role-reversal (e.g., alternately switching between dominant and submissive positions) are Fp behaviors that have evolved to facilitate the acquisition of skills for behaving fairly as adults. For humans, Bekoff (2001) has suggested that Fp may be the basis not only for fair behavior (Fb) but also for morality. By focusing on self-handicapping and role-reversal in juvenile play and its implications for morality, Bekoff (2001) argued for an equitability interpretation of fairness (i.e., fairness as the equitable distribution of resources or treatment of others).

From an evolutionary perspective, the hypothesis that Fp is beneficial for acquiring skills for behaving equitably and possibly for acquiring moral behavior as adults is problematic. While previous research (Durand and Schank, 2015) demonstrated that social play could evolve by facilitating adult cooperation, it is not clear that these theoretical results extend to fairness as equitable behavior. Fairness and cooperation are closely connected but they are not synonymous. Consider, for example, two hunters who cooperate to hunt elk instead of individually hunting for rabbits or squirrels. Taking down an adult elk provides considerably more meat than either hunter could bring home hunting individually for rabbits and squirrels. Hunting cooperatively for an elk provides a synergistic payoff greater than individual payoffs from hunting rabbits and squirrels. For cooperation to persist, the payoff of cooperative hunting must benefit both hunters, but this does not imply that the distribution of the elk has to be fair in the sense of an even distribution (assuming the hunters have the same abilities, needs, and contributed the same effort). Even though both hunters contributed equally to elk the hunt, if one takes 75% of the elk leaving 25% for the other hunter, it is still in the interest of the hunter receiving the lesser amount to participate in future cooperative hunts if 25% of a stag is more meat than a couple of rabbits or squirrels. That is, if the expected payoff from cooperating is greater than the expected payoff for not cooperating (even when the distribution of gains is not fair), unfair cooperation will be favored by evolution. This example suggests that evolution of cooperation does not guarantee the evolution of fairness.

A model of the evolution of Fp in juveniles must show that fairness is selected for as adults. Dugatkin and Bekoff (2003) developed a game-theoretical model aimed at showing that Fb in juveniles could stably evolve by promoting fairness in adults. Their model consisted of two-developmental stages. As young animals, individuals either engage in Fp or they do not (NFp). As adults, they either engage in Fb or they do not (NFb). This results in four possible strategies Fp/Fb, Fp/NFb, NFp/Fb, and NFp/NFb and each pairwise combination of strategies (e.g., in strategy pair Fp/Fb, Fp/Fb, and an individual plays another with the same strategy) had a distinct probability of obtaining a resource *R*. Fb learned during juvenile Fp is represented by the strategy Fp/Fb and Dugatkin and Bekoff (2003) assumed that when Fp/Fb plays itself, that fairness is a 50:50 split of the resource. When Fp/Fb plays an unfair strategy (i.e., Fp/NFb and NFp/NFb), the resource split favors the unfair strategy. In addition, their model assumed that Fp/Fb playing itself had the highest probability of obtaining a resource. They found that Fp/Fb is a pure evolutionary stable strategy as long as the payoff when playing against Fp/NFb is not greater than or equal to the payoff for Fp/Fb when playing itself. However, this result depends on the assumption that the total payoff of Fp/Fb against itself is higher than any other combination. Without this synergistic assumption, fairness—as a 50:50 split of the resource—cannot evolve.

It might be expected that social play in animals would display a 50:50 win-loss ratio in social play encounters as assumed in Dugatkin and Bekoff (2003) model, but there are many factors that could affect equitability. Empirical studies have shown that social play can deviate markedly from a 50:50 win-loss ratio due to factors such as age, sex, dominance and species differences (e.g., Pellis et al., 1993; Biben, 1998; Bauer and Smuts, 2007; Cordoni and Palagi, 2011), and even in similarly matched juveniles, role reversals occur at a rate of around 30% (Himmler et al., 2016; Pellis and Pellis, 2016). Indeed, the similarly below 50% level of role reversals in juveniles, reflecting a degree of reciprocity in play, is present across species that use very different behavioral mechanisms to ensure that some reciprocal exchanges occur during social play (Pellis and Pellis, 2017). Importantly, though, it should be noted that while the reciprocity in such play need not be equitable, excessive deviation toward one partner persistently gaining the upper hand leads to unstable play partnerships. Typically, the individual that is too overbearing becomes ostracized from the potential play partners in the group (e.g., Wilmer, 1991; Suomi, 2005).

While empirical studies provide some evidence that social play in juveniles can be fair (as described above), we still have no evidence that Fb in adults can be beneficial in itself. Because fairness does not imply a synergistic gain, it is difficult to conceive of how fairness could be selected *for*. To illustrate this point consider a group of hunter-gatherers. Assume all are exactly the same in ability, needs, and effort they put into obtaining resources. Fairness in this case implies an even distribution of resources because no individual is entitled to any more than the others because their abilities, needs, and effort are the same. There are two strategies these hunter-gatherers can use: selfish and fair. Individuals using a selfish strategy keep the resources they obtain each day while individuals following a fair strategy pool their resources for an even distribution at the end of the day with others who also adopt a fair strategy. There is no synergistic gain in pooling. Assuming individuals using both strategies are equally successful, *p*, in obtaining a resource, *R*, on a given day, the expected payoff over time for individuals using the selfish strategy is *pR*. For fair individuals who pool their resources, the total pool is *mpR*, where *m* is the number of individuals using the fair strategy. After equitable division (*mpR*/*m*), each fair individual’s expected payoff is also *pR*. Thus, the long-term expected payoffs for individuals adopting either fair or selfish strategies is exactly the same, *pR*. There is apparently no clear benefit for Fb as adults and if Fp as juveniles is costly, then there appears to be no theoretical basis for the evolution Fp as a learning or skill refining context for adult fairness and moral behavior.

Expected payoff is not the only way to characterize payoffs for fair and selfish strategies. There is also variance in payoffs among individuals adopting fair or selfish strategies. Individuals adopting the fair strategy pool and equitably divide their resources each day. Daily variance in payoffs for a group of fair individuals is easy to calculate at the end of the day, it is zero. For selfish individuals, although their expected payoff in the long run is *pR*, on each day they only have a probability *p* of success. Some selfish individuals will succeed in obtaining *R* resources, but (1 – *p*) other individuals will fail to obtain any resources. The expected variance among individuals adopting the selfish strategy can be calculated on the assumption that for a group of *m* individuals, *pm* of them will obtain a resource and (1 – *p*)*m* of them will fail yielding Eq. 1.

(1) $$\begin{array}{c} \mathrm{var}(R,p)=(1-p)pR^{2}\mathrm{var}(R,p)=(1-p)pR^{2} \end{array}$$

For example, if *R* = 40 units and *p* = 0.0875, variance is 127.75. Thus, even though there is no difference in the expected long-term payoff to either fair or selfish strategies, there is a large difference in daily variance in payoffs. Could differences in payoff variance play a role in fitness differences? If so, then it may be possible to show that the apparently worst-case scenario for fairness as equitability (i.e., even distributions of resources) has fitness benefits.

To illustrate how payoff variance may play a role in fitness, consider the dictator game. The dictator game is a simple 2-person game in which one player, the dictator, decides how to divide a resource with a second player. Since the second player has no leverage, the rational decision for the dictator is to keep all of the resource and give nothing to the second player because the second player has no counter strategy. However, numerous empirical studies have found that dictators give on average 30% to the other player (Engel, 2011). Thus, while it is surprising that dictators behave far more equitably than predicted they also do not, on average, evenly divide resources. Schank et al. (2015), using an agent-based model, showed that when population structure emerges from agent aggregation, clusters or groups of agents that more equitably distribute resources produce more offspring than those that do not. According to their analysis, the advantage of more equitable distributions of resources is due to the more efficient conversion of resources into offspring when there are constraints on the flow of resources to offspring. Interestingly, the sharing of resources need not be an even split to gain the benefit of more efficient conversion of resources into offspring.

In this paper, we developed an approach along the lines of Schank et al. (2015) to model the evolution of fair social play. Our model aimed to investigate the evolutionary plausibility of social Fp having its adaptive benefit in facilitating the learning of adult Fb. Our model, like Dugatkin and Bekoff (2003), has two developmental stages: a juvenile stage in which agents can engage in social Fp with mortality cost, *c*, which is the probability of dying when engaged in social play (e.g., killed by a predator due to increased exposure from playing). Our model is also similar to Auerbach et al. (2015) in that it is based on asexual reproduction, involving a single gene, but differed in that they modeled asocial play. As adults, agents forage for resources, *R*, at each time step with probability *p* of success. Agents that have learned to play fairly as juveniles pool their resources with other fair agents (if any) and then evenly divide the pooled resources at the end of each simulation step. Agents that have not learned to be fair, simply keep the resources (if any) they obtain. We hypothesized that Fp would evolve—even with juvenile mortality due to social play—when there is considerable variance in foraging for resources (i.e., likelihood of obtaining a resource is relatively low but the value of the resource is relatively high, for example, mimicking foraging in hunter-gather societies, see discussion). We show that Fp can evolve under these reasonable conditions and that our model can serve as a first step in the development of a rigorous theory of the evolution social play.

## Model and Simulation Methods

Our aim was to develop a generically realistic model of the evolution of social play rather than a model for a particular species. By generic we mean a model that represents very general biological properties of animal social systems in which social play can evolve. By developing a generic model of Fp, this can facilitate the future extension of this model to specific species and social play systems. Although our model is not strictly speaking a game-theoretical model (i.e., fair and selfish strategies do not directly affect the payoffs of each other), it does share features in common with other game-theoretical models using agent-based modeling (for a recent review see Adami et al., 2016).

In our model, animals reproduce and invest some of their resources into their offspring. There are many ways organisms can reproduce, but we have selected a very simple mode of asexual reproduction with a single gene for social play. Variation is constantly introduced by random mutation of the play gene (i.e., play genes mutate to an *on* or *off* state depending on the prior state of a parent) at a low frequency. We assumed agents have an average lifespan with Guassian variation about the mean to model the myriad causes of death without modeling these causes in specific detail. Finally, we assumed that agents live in small social groups and that juvenile agents can play with other juveniles in their group.

Development is simplified to consist of two stages, juvenile and adult, similar to the assumption made by Dugatkin and Bekoff (2003). During the juvenile stage, agents can engage in social play with a potential cost of death while learning to behave fairly as adults. As adults, all agents accumulate resources if they have learned to behave fairly, they can share their resources on each round of play with other fair agents. Resources are converted into offspring by reproduction. An adult agent can reproduce if it has accumulated sufficient resources and a minimum “gestational” period has occurred between reproductive events. To our knowledge, introducing a delay between reproductive events has never been done in an evolutionary model, but is a generic characteristic of all multicellular organisms. Based on Schank et al. (2015), delays between reproductive events should constrain the flow of resources into offspring resulting in fitness benefits for fairness.

### Model Details

Adult agents are assumed to live in groups with their offspring. The number of groups, *n*, in a population is limited to a maximum of *G* groups with a total maximum population size of *K*. For example, if *G* = 50 and *K* = 1000, then the maximum average group size is 20 adults. When all the members of a group die, the group is extinguished. When a group reaches it fission size *f* and the number of groups is less than *G*, the parent group fissions producing an offspring group by randomly selecting *g* adult members from the parent group to form the offspring group.

Agents have two developmental stages: juvenile and adult. Juvenile agents engage in social play. Adult agents forage for resources to reproduce. The juvenile stage is *j* steps long and during this period, agents can engage in social play if their social play gene is *on*, otherwise they do nothing (below we will refer to the play gene in the *on* state as the *play gene*). Juvenile agents can learn to behave fairly as adults if they engage in at least *α* bouts of Fp. Only one bout of play with another juvenile can occur on a given simulation step. Each bout of Fp comes with a potential cost, *c*, of mortality. That is, there is a random chance with probability *c* that an agent dies during a bout of play. Agents that do not play suffer no mortality cost. A juvenile agent finds a play partner by randomly querying (analogous to directing a play invitation signal) other juvenile agents in its group until it finds another juvenile that will play (i.e., has the play gene) or until it has queried all juveniles in its social group and found none that will play. If a juvenile agent does not find any other juvenile agents to play with, it does not play. Thus, when the frequency of the play gene in a population is low, some agents with the play gene may not learn to play fairly but also will not suffer the cost *c* of engaging in Fp.

When a juvenile reaches the *j*th simulation step after birth, it becomes an adult and enters its social group if the total number of adult agents in the population is less than *K*. If there are *K* or more adult agents in the population, then the juvenile agent dies. This method holds the number of adult agents in the population to no greater than *K* by assuming juvenile mortality occurs at a higher rate than adult mortality, which is biologically reasonable (Caughley, 1966). This method introduces no bias into the simulation at the juvenile stage because other than mortality due to play, whether a juvenile becomes an adult is entirely random with respect to *K*.

During the adult stage, reproductive output is dependent on resource acquisition, which implies that the more resources an agent obtains, the greater its reproductive output. Adult agents forage for resources, *R*, on each simulation step with probability *p* and so the expected payoff for each agent is *pR* (e.g., if *R* = 40 units and *p* = 0.0875 the expected payoff would be 3.5 units over time). The resource *R* is the mean of the resources agents can obtain on a given step and the quantity of the resource obtained is *R* plus a random Gaussian deviate with standard deviation *SD_R_* = 0.1*R* (10% of the mean resource). Agents that learn to be fair by engaging in social play and those that do not, have the same success rate, *p*, of obtaining resources, *R*. Fair adult agents pool their resources with other fair adult agents in their social group on each simulation step and then divide the pooled resources at the end of each simulation step. Because fair agents pool and then divide their resources on each round of play, their expected payoff is exactly the same as selfish agents, *pR*. Thus, there is no apparent reproductive advantage to fair agents pooling their resources based on expected payoffs.

Adult agents can reproduce when they have accumulated resources sufficient to reach or surpass a threshold *T*. The timing between reproductive events is constrained by a reproductive delay *d*. That is, if an agent reproduces at step *t* then the earliest it can reproduce again is *t* + *d*. The reproductive delay, *d*, can be interpreted, for example, as a fixed gestational period. Reproductive delays constrain the number of offspring that can be produced in a lifetime. Unlimited resources cannot result in unlimited reproduction in this model.

**FIGURE 1:**
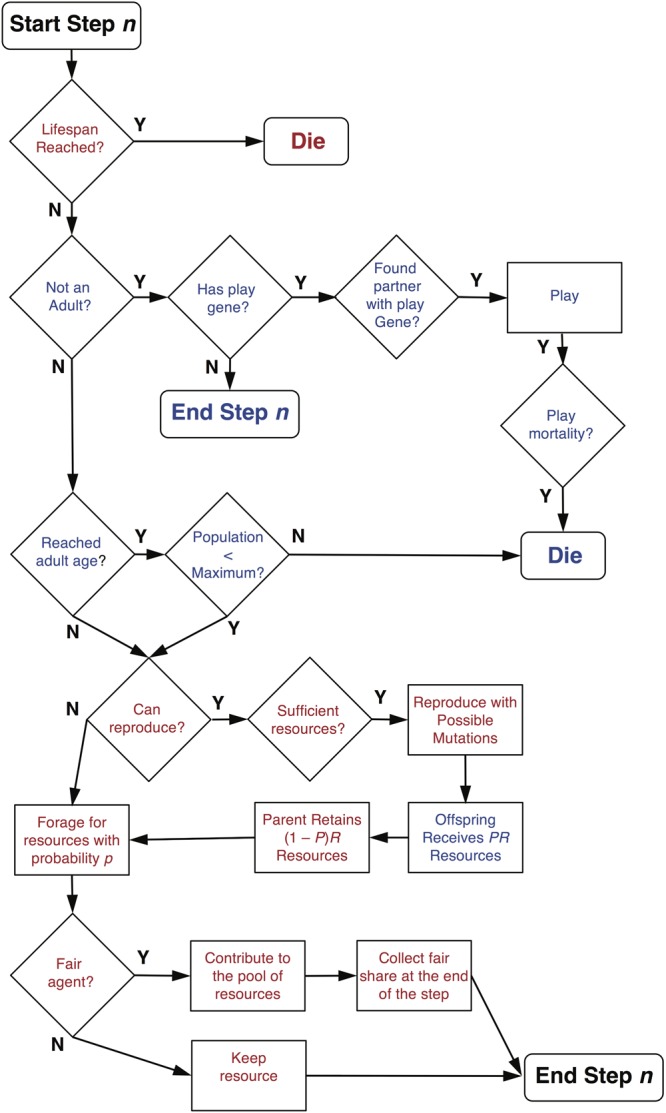
Decision and event diagram for agents on each step of a simulation. Text in red indicates decisions and events for adult agents and text in blue indicates decisions and events for juvenile agents. Note that fair adult agents only receive a share of the common resource pool after all agents have contributed on a given step.

Agents have only one gene, which is a social play gene that is in one of two possible states: *on* or *off*. Offspring inherit the state of their play gene from their parent, but the state can be flipped to the opposite state by mutation at rate *r*. A parent contributes a portion *P* of its accumulated resources (i.e., the total amount of resources it has accumulated up to that step) to its offspring and keeps (1 – *P*) resources. When an agent is born, it is assigned a lifespan, which is a random integer composed of the mean lifespan *l* plus a randomly generated integer (±) drawn from a Gaussian distribution with standard deviation *SD_l_*. When an agent reaches the end of its lifespan, it dies and is removed from the simulation including its current juvenile offspring. The underlying assumption is that the juvenile agents are dependent on the parent and do not survive a parent’s death. Alternatively, it could have been assumed that dependent juvenile offspring survive the death of a parent. For this model, since adult death does not depend on the resources collected, but rather a randomly assigned death date, the choice of assumption is not crucial. If, however, the lifespan of an agent depends on its behavior, then such assumptions do matter (see **Figure 1** for an agent’s decisions and possible events during s simulation step).

### Simulations

The parameter values used in all simulations are listed in **Table 1**, initial conditions listed in **Table 2**, and the parameter sweeps used in the two experimental sets of simulations are listed in **Table 3**. Control simulations were run, which differed from the experimental simulations in that agents did not learn fairness from engaging in Fp as juveniles. In this model, the dependent variable is the frequency of the Fp gene (i.e., the play gene is in the *on* state). Because the frequency of the play gene in a population correlated, as expected, very closely with the frequency of fair adults in a population, the frequency of the Fp gene is also a very accurate proxy for the frequency of adults that learned to behave fairly in these simulations.

**Table 1:** Fixed parameters, values, and descriptions.

Parameters	Values	Description
*K*	1000	Maximum number of agents in a population.
*G*	50	Maximum number of groups in a population.
*f*	40	Fission size of groups: when reached, offspring group consists of *g* randomly drawn adults from the parent group.
*g*	20	Offspring group size.
*j*	50	Length of juvenile stage in simulation steps.
*α*	5	Number of bouts of play required to learn to be fair as an adult.
*P*	0.5	Parental investment
*T*	100	Reproductive threshold for producing one offspring
*r*	0.01	Mutation rate for flipping a play gene on or off.
*l*	150	Average lifespan.
*SD_l_*	25	Lifespan standard deviation.

**Table 2:** Initial conditions.

Parameters	Values	Description
*R_T_*	[50, 150]	Initial total resources an agent has at the beginning of a simulation. A uniform random real number drawn from the indicated range.
*A*	[50, 150]	Initial age of an agent at the beginning of a simulation. A uniform random integer drawn from the indicated range.
*G*	50	Initial number of groups.
*n*	20	Initial number of agents in each group.
*F*_0_	0.05	Initial frequency of play genes in the population.
*τ*	25000	Simulation steps.

**Table 3:** Parameter sweeps.

Parameters	Values	Description
**Common to all simulations**		
*P*	0.1, 0.3, 0.5, 0.7	Parental investment
*c*	0, 0.0005, 0.001, 0.0015, 0.002, 0.00225, 0.003	Cost of social play: probability of dying during each social play episode and produced mortality costs of 0, 1.9, 3.6, 5.4, 7.0, 8.0, and 10% on average.
*d*	0, 1, 2, 3, …, 50	Delay between reproductive events
**First set of simulation**		
*R*	10, 20, 30, 40, 50	Resource quantity obtained with 10% SD Gaussian random noise.
*p*	0.35, 0.175, 0.11667, 0.0875, 0.07	Corresponding to values of *R*: foraging rates (probabilities) of obtaining *R* on a given round.
**Second set of simulations**		
*R*	40	Resource quantity obtained with 10% SD Gaussian random noise.
*p*	0.05, 0.0625, 0.075, 0.0875, 0.1, 0.1125, 0.125	Corresponding to the value of *R*: foraging rates (probabilities) of obtaining *R* on a given round.

Before running the main simulations reported here, we ran preliminary simulations to determine how many simulation steps were required to reach equilibrium frequencies of the play gene. Based on these simulations, we estimated that 10000 simulations steps were required to reach estimated equilibrium frequencies. We then ran all simulations for an additional 15000 steps giving a total of 25000 steps. This allowed us to calculate the frequency of the play gene based on the number of agents born with the play gene over the total number of agent born in the interval 10000 to 25000 steps. Based on these calculations, frequency estimates of the play gene were based on at least 21000 agents for each simulation experiment. For each set of parameter conditions, we ran 20 simulation experiments. Thus, the play gene frequencies reported for each set of parameter conditions were based on at least 420000 agents and so the frequency results reported here are based on very large numbers of observations.

The theoretically interesting parameters in this model are parental investment, *P*, the average resources *R* obtained in a successful foraging bout, the foraging success rate *p*, and the juvenile mortality play cost, *c*. Variance in foraging was hypothesized to create the opportunity for selection on adult fairness. We generated different levels of variance in two ways. First, we held expected foraging success *pR* = 3.5, constant (the expected payoff should be relatively small so that a substantial number of simulation steps are required to accumulate sufficient resources to reproduce) and then systematically varied combinations of *p* and *R* (see **Table 3**, first set of simulations). Second, we held *R* = 40 constant and varied *p* to produce a range of expected payoffs, *pR* (see **Table 3**, second set of simulations). The mortality cost, *c*, is the probability that a juvenile agent dies as a result of engaging in social play. We investigated different values of *c* (see **Table 3**) that generated different percentages of juvenile mortality due to play. Finally, we simulated four levels of parental investment *P* (see **Table 3**) to assess the effect of parental investment on the evolution of the play gene.

More precisely, for the first set of simulations, the expected payoff *pR* was held constant at 3.5 and we investigated a range of payoffs *R* = 10 to 50 with increments of 10 (see **Table 3**). For each expected payoff, we ran 20 simulations for reproductive delays of 0 to 50 in increments of 1 for the seven mortality conditions. This resulted in 5 × 51 × 7 = 1785 sets of simulations for a total of 20 × 1785 = 35700 simulations. For each of four parental investment values, we repeated these 35700 simulations for a total of 142800 simulations. For the second set of simulations, we held constant at *R* = 40 and varied the foraging success rate, *p*, of obtaining *R* such that the expected payoffs ranged from 2 to 5 in increments of 0.5 (see **Table 3**). For each expected payoff, we again ran 20 simulations for reproductive delays of 0 to 50 in increments of 1 for the seven juvenile mortality rate conditions. This resulted in 7 × 51 × 7 = 2499 sets of simulations for a total of 20 × 2499 = 49980 simulations. For each of the four parental investment values, we repeated these 49980 simulations for a total of 199920 simulations. Thus, we ran a total of 342720 simulations, which lasted up to 25000 steps each with populations of 1000 agents.

We also ran control simulations, which were exactly the same as the experimental simulations except that agents did not learn adult fairness from juvenile Fp. Control simulations were required because the expected payoffs for fair and selfish agents were the same and in the absence of selection, the frequency of the play gene should evolve to 50% when there is no mortality cost for social play. A positive mortality cost of juvenile agents engaging in social play but not learning to behave fairly as adults does not guarantee that the frequency of play gene will drop to zero. This is because at low frequencies, there will be too few if any juvenile agents in a small group that have the play gene. Thus, at low frequencies, the play gene will suffer little if any mortality cost due to social play and mutation will continue to reintroduce the play gene at a low rate (see **Table 1** for mutation rate).

Mortality cost, *c*, is the probability of dying when engaged in social play. Values of *c* were selected to generate a range of mortality rates ranging from 0% to just over 10% mortality in juveniles engaged in social play. Because of the complexities of how often juvenile agents actually play, mortality percentages can only be calculated by recording how many juvenile agents die during a simulation. Different values of *c* were used (see **Table 3**), which generated mortality percentages, which varied among different simulations sets. For example, when the probability *c* of dying during a play bout was *c* = 0.003, this typically resulted in a 10% mortality rate. In some sets of simulations, the record mortality may have been 10.2% and in others 9.9%.

For simulations with parental investment of *P* = 0.1, populations often went extinct for large reproductive delays (*d* > 45). Agents, on average, live for additional 100-time steps after they become adults. Reproductive delays greater than 45 steps imply that adults can reproduce at most twice. When parental investment, *P* = 0.1 is very low, new adult agents may require more than 25 steps to accumulate sufficient resources, reducing the average individual reproductive rate below sustainable levels when combined with positive juvenile mortality costs. Thus, in the results reported below, the average evolved frequency of the play gene for parental investment of *P* = 0.1, were average for values of *d* ranging from 0 to 45.

The agent-based model was written in Java using the agent-based modeling library provided in MASON (Luke et al., 2005). All simulations were run on computers using Scientific Linux^1^.

## Results

**FIGURE 2:**
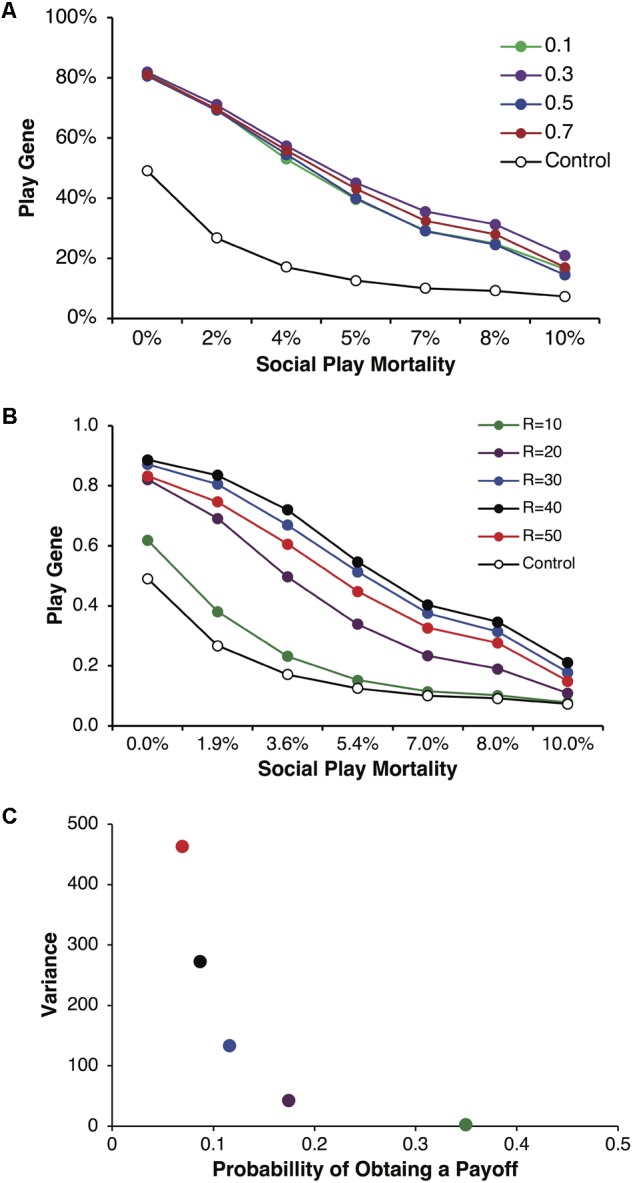
Mean frequencies of the play gene plotted against mortality costs for four different values of parental investment, *P* = 0.1, 0.3, 0.5, and 0.7 **(A)**. Expected payoffs were held constant at *pR* = 3.5 by multiplying values of *R* with values of *p*. Mean play-gene frequencies for parental investment of *P* = 0.5 only and plotted by the expected quantity of *R* = 10, 20, 30, 40, and 50 **(B)**. Although expected payoffs were held constant, varying *R* and *p* generated different degrees of expected resource variance on each round **(C)**. Resource variance was calculated using Eq. 1 for values of *p* and *R* and then plotted against the probability *p* of obtaining a resource payoff on a given round of play. The combination of lower probability of payoff and higher resource quantity (compare colors in **A** and **B**) generated considerably different levels of variance. For *R* = 10, *p* = 0.35 (green), variance was very low and the play gene evolved to frequencies barely above chance and only for the lowest social play mortality costs. In contrast, for *R* = 40, *p* = 0.0875 (black), variance was high and the play gene stably evolved well above chance levels even for the highest rate of social play mortality. However, the social play gene did not evolve monotonically with increasing variance. The values *R* = 50, *p* = 0.07 (red) produced the highest variance but not yield the highest frequency of play genes.

We found that the play gene evolved to frequencies greater than in control simulations across a wide range of conditions as the variance in payoffs increased. **Figure 2A** illustrates the evolved frequencies of the play gene for different values of parental investment including corresponding control simulations. Each point is averaged over reproductive delays and different values of *p* and *R*. For all values of parental investment, the evolved frequency of the play gene was above the control simulation values. For these simulations, parental investment had a relatively small effect on the overall evolution of Fp. **Figure 2B** illustrates results for a representative parental investment of *P* = 0.5. Simulation results were again averaged over reproductive delays, but the values of *p* and *R* were varied to produced different degrees of variance (see **Figure 2C**) while holding *pR* = 3.5 constant. As variance (calculated using equation 1), increased with greater values of *R* (**Figure 2C**), the stably evolved frequency of the play gene increased until *R* = 50, where it was slightly lower than for *R* = 30, 40. The lowest variance occurred for *R* = 10, as expected, and the frequency of the play gene barely evolved above chance (**Figure 2B**).

**FIGURE 3:**
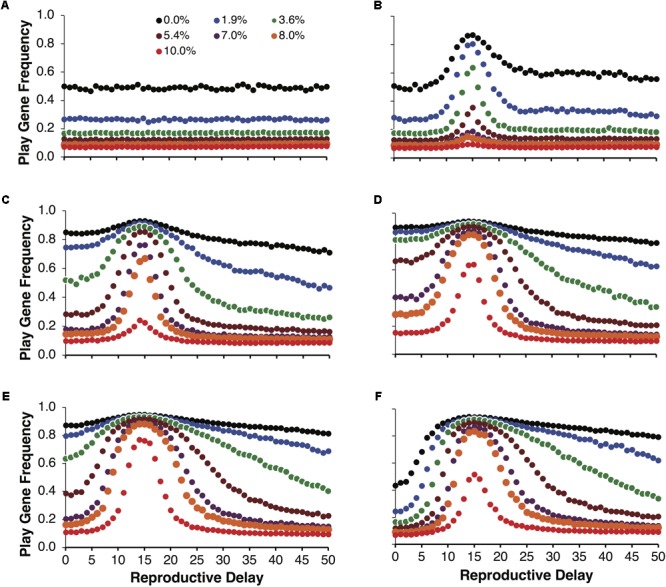
Mean play gene frequencies after 25,000 simulated steps for the same expected payoff of 3.5 in **Figure 2** but with different degrees of variance among non-fair agents. Control simulations over reproductive delays **(A)**. The remaining figures provide a finer-grained analysis of play-gene frequencies as a function of reproductive delays. Panels **(B–F)** correspond to different resource variances, respectively. When there was no mortality cost for social play, the frequency of the play gene typically evolved to about 80% except for the lowest variance condition [var(10,0.35) = 22.75; **B**] in which the play gene only evolved to about 80% at the reproductive delay, d = 15. As mortality due to social play increased, the evolved frequency of the play gene rapidly decreased except close to the reproductive delay, d = 15 **(B–F)**. Play gene evolution was most favored for the next to highest variance condition (R = 40, **E**). In panel **(E)**, near the d = 15 delay, the play gene is maintained at over 70% even with 10% mortality.

**FIGURE 4:**
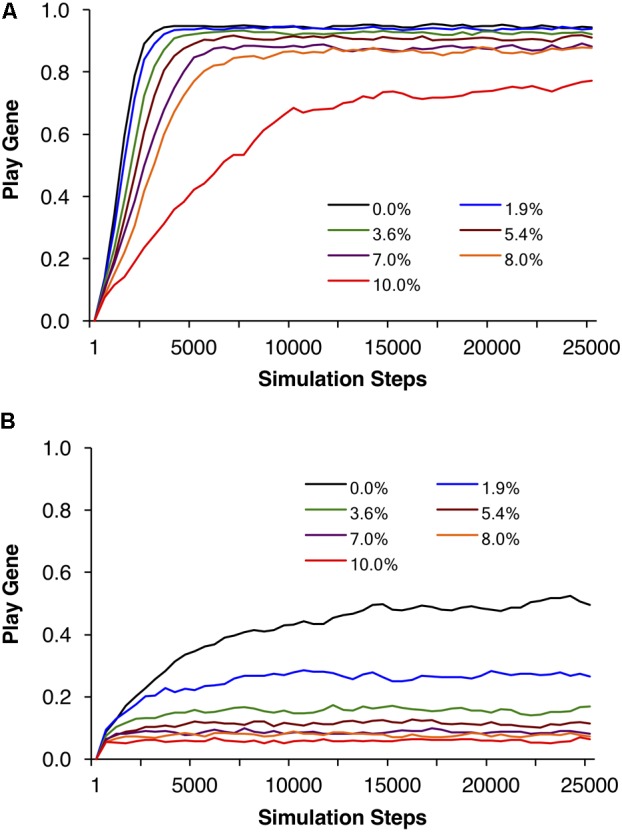
Example individual simulations for *R* = 40, foraging success rate of *p* = 0.0875, and expected payoff of *pR* = 40 × 0.0875 = 3.5. Parental investment was set to *P* = 0.5 and the reproductive delay was set to *d* = 15. Each trajectory represents the mean of 20 simulations of 1000 adult agents each. Seven simulations with different percentages of social play mortality are depicted in **(A)**. Corresponding control simulations in which agents engage in social play as juveniles but do not learn to be fair as adults are depicted in **(B)**. In all but the highest mortality condition, populations evolved to over 80% fair agent **(A)**, but even for 10% mortality, the mean number of agents that evolved the play gene was over 70%.

**Figure 3** illustrates the same simulations as in **Figure 2A**, but not averaged over reproductive delays. **Figure 3A** shows the control simulations, which as expected did not vary as a function of reproductive delay. **Figures 3B–F** plot the evolved frequencies of the play gene as a function of reproductive delay. These figures also illustrate the intensity of selection for the Fp gene as a function of reproductive delay. We see that different reproductive delays interact with expected payoffs so that the intensity of selection for the play gene is very high for a narrow range of *d*. For these sets of simulations, the peak intensity of selection occurred with a reproductive delay of 15. At peak selection intensity, high frequencies of the play gene could be maintained in the face of juvenile mortality ranging from 8 to 10% (e.g., **Figure 3E** with *R* = 40 and *p* = 0.0875). In contrast with the averaged results in **Figure 2**, even for the lowest variance condition var(10, 0.35) = 22.75, the play gene evolved to 80% at *d* = 15 when social play mortality was 1.9% (**Figure 3B**). In **Figure 3E**, for var(40, 0.0875) = 127.75, the play gene evolved to 76% at *d* = 15 even with a 10% juvenile mortality rate. (see **Figure 4** for the evolution over time steps of the simulations illustrated **Figure 3E** for *d* = 15).

**FIGURE 5:**
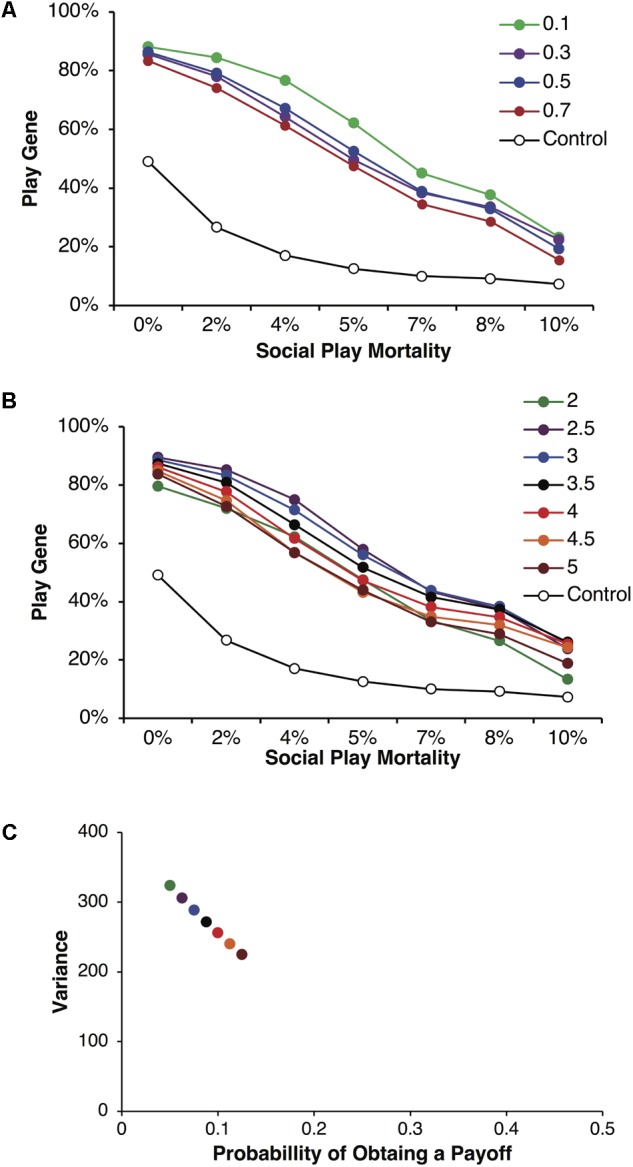
Mean frequencies of the play gene plotted against mortality costs for four different values of parental investment **(A)**. The points plotted in are averages of sets of simulations with *R* = 40 and different foraging success rates, *p*, yielding expected payoffs ranging from 2 to 5. Mean play-gene frequencies for parental investment of 0.5 with expected payoffs, *pR*, ranging from 2 to 5 are plotted in **(B)**. A plot of the expected variances for each expected payoff in **A** and **B (C)**. Panel **A** illustrates that social play can robustly evolve even under the highest social play mortality conditions, averaging equilibrium values for individual sets of simulations provides only the crude depiction of the complexity of multilevel evolutionary process (see **Figure 6**).

In **Figure 5**, we held the mean payoff constant at *R* = 40 but varied the foraging success rate *p* and thus the expected payoffs, *pR*, varied from 2 to 5. In **Figure 5A**, parental investment *P* was varied and each point is averaged over reproductive delays and different expected payoffs. In these simulations, the play gene evolved to higher frequencies than the control simulations for all values of parental investment. Under low parental investment (*P* = 0.1), the play gene evolved to the highest frequencies. Parental investment of *P* = 0.5 was approximately in the middle, and the play gene evolved to the lowest levels when *P* = 0.7. **Figure 5B**, illustrates the results for parental investment of *P* = 0.05, with the results for each value of the expected payoff *pR* plotted individually. In all of these simulations the play gene evolved well above control simulation frequencies for all levels of juvenile mortality. All simulations have very similar results (due to smaller range in resource variances, **Figure 5C** as compared to **Figure 2C**) even though there was considerable range in expected payoffs (2 to 5; **Figure 5B**).

**FIGURE 6:**
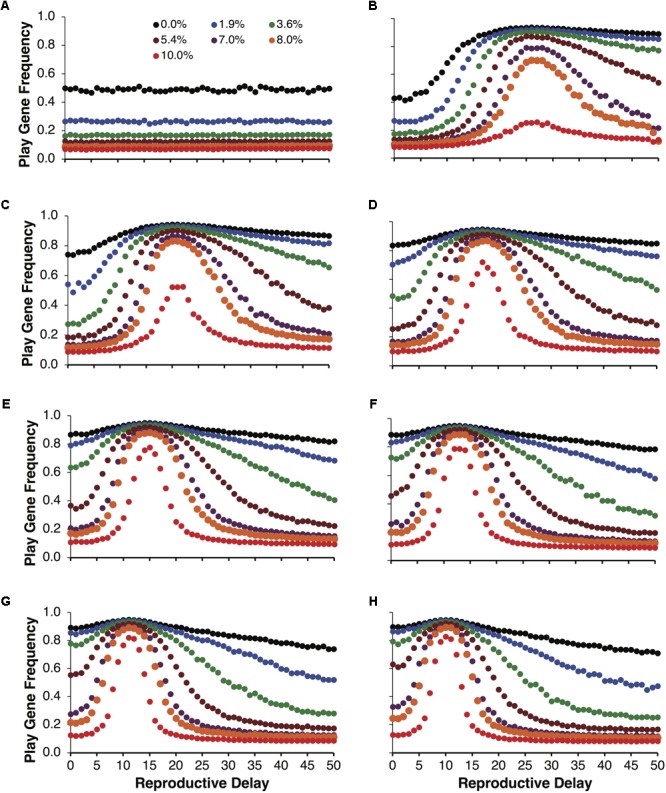
Mean play gene frequencies after 25,000 simulated steps for expected resource payoffs depicted in **Figure 5B** but not averaged over reproductive delays, *d*. The frequency of play genes in the control condition when juvenile agents engage in play but did not learn to play fairly as adults plotted as a function of juvenile play mortality ranging from 0.0% to 10.0% **(A)**. The remaining figures represent the frequency of social play when agents learned to play fairly as adults for expected payoffs ranging from 2 to 5 in increments of 0.5 **(B–H)**.

As with **Figure 3**, examining the evolution of play gene with respect to reproductive delays paints a more complex structure. In **Figures 6B–H**, the evolution of the play gene evolves above chance levels (**Figure 6A**) for low to moderate juvenile mortality. In **Figure 6B**, when the expected payoff *pR* = 2 was the lowest of all the conditions, short reproductive delays (*d* = 0, …, 5) resulted in the evolution of play gene frequencies at the level of chance (cf. **Figure 6B**). However, as the reproductive delay, *d*, increased beyond *d* = 5, play gene frequencies began to increase. For example, for the 8% mortality condition, play gene frequency in population reached 70% with reproductive delays, *d*, of 26 to 27 (**Figure 6B**). Interestingly, **Figures 6B–H** illustrate that the intensity of selection for Fp is a function of the reproductive delay for expected payoffs ranging from 2 through 5 in increments of 0.5 (**Figures 6B–H**). In **Figures 6B–H**, the delays resulting in peak selection intensity occur at d = 26 (**Figure 6B**), 20 (**Figure 6C**), 17 (**Figure 6D**), 14 (**Figure 6E**), 13 (**Figure 6F**), 11 (**Figure 6G**), and 10 (**Figure 6H**).

**FIGURE 7:**
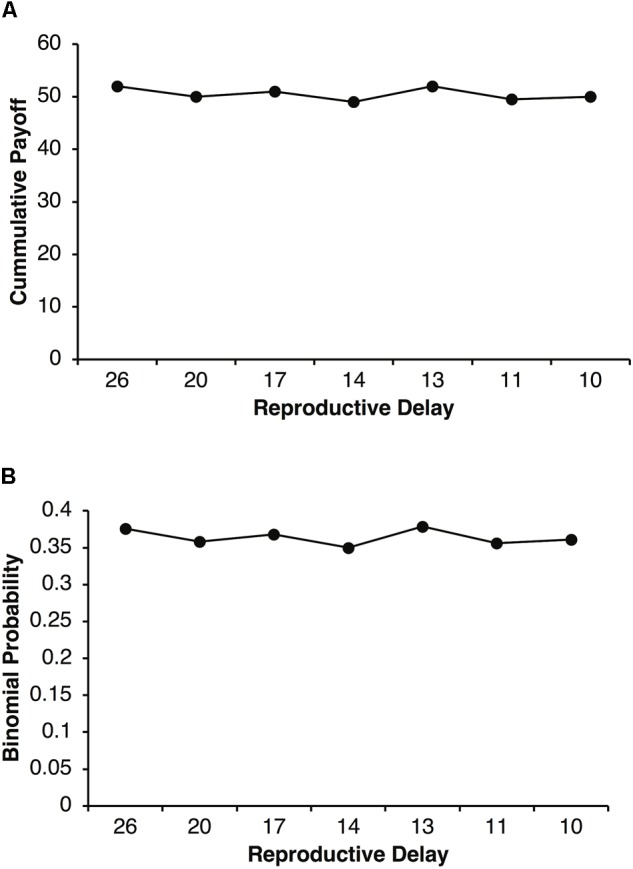
Plot of the expected cumulative payoff as a function of reproductive delay *d* and expected payoff *pR* **(A)**. Reproductive delays were *d* = 26, 20, 17, 14, 13, 11, and 10 with expected payoffs of *pR* = 2, 2.5, 3, 3.5, 4, and 4.5. For example, the expected cumulative payoff for a reproductive delay of *d* 10 with *pR* = 5, is 50. Binomial probability of an agent obtaining two or more reproductive payoffs of *R* = 40 during a reproductive delay *d* **(B)**.

The increased selection for adult Fb that peaks around specific values of *d* in **Figures 3, 6**, can be explained in terms of parental investment, *P*. **Figure 7A** plots the cumulative expected payoffs during reproductive delays of *d* = 26, 20, 17, 14, 13, 11, and 10 with the corresponding expected payoffs per round of *pR* = 2, 2.5, 3, 3.5, 4, 4.5, and 5. In each case, *dpR* is close to 50 (e.g., for *d* = 10, *p* = 0.125, and *R* = 40, *dpR* = 50). With a reproductive threshold of *T* = 100 units of resources and parental investment of *P* = 0.5, a parent is expected to retain about 50 units of it resources after a reproductive event. Thus, about 50 units of resources are required to reproduce again. A fair agent with a *pR* = 2.5 will require, on average, about 20 rounds of foraging to accumulate about 50 units of resource where as a fair agent with a *pR* = 5 will only require about 10 rounds of foraging. Selfish agents have the same cumulative expected payoff except that their payoffs come in chunks of size *R*. Thus, during reproductive delays there is a higher probability that selfish agents will not accumulated the required resources during the delay period (i.e., *t* + 1 to *t* + *d*). For example, a selfish agent requires at least two payoffs of *R* = 40 during a reproductive delay *d* to reach the reproductive threshold. The binomial probability of a selfish agent achieving this threshold in *d* rounds is less than 0.4 as illustrated in **Figure 7B**. On the other hand, fair agents have a slow but steady accumulation of payoffs that on average achieves the reproductive threshold in *d* simulation steps.

## Discussion

We found that juvenile Fp could evolve by facilitating the acquisition of skills for equitable behavior in adulthood. This provides theoretical support for the working hypothesis that adult fairness could be beneficial and, as Groos (1898) long ago proposed, a benefit of social play comes from learning specific adult social skills as juveniles, in this case fairness. These results also provide support for the more controversial idea proposed by Bekoff (2001) and Palagi et al. (2016) that young animals engaging in Fp acquire skills needed to behave fairly as adults. Our results indicate that Fp behaviors, such as self-handicapping (e.g., an individual not biting as hard as it can) and role-reversal (e.g., alternately switching between dominant and submissive positions), could have evolved to facilitate the acquisition of skills for behaving fairly as adults.

In our model, adult agents could either keep what they foraged or pool it with other fair agents and then distribute pooled resources evenly among themselves after each round of foraging. Selfish and fair agents had the same expected payoffs but variance in accumulated resources was less for fair agents. This allowed resources to flow more efficiently into the production of offspring. By imposing a “gestation” period (reproductive delay) on agents, we found that this greatly affected the intensity of selection for fairness even in the face of high juvenile mortality costs (see **Figures 3, 6**). Such constraints enhance the advantages of fairness because unfair agents cannot convert all of their resources into offspring due to “gestational” delays. In other words, assuming no constraints on the rate of reproduction is equivalent to assuming that by feeding a female rat twice as much will either double her litter size or cut the gestation period for her pups in half. Neither are biologically plausible or possible assumptions but they are implicitly assumed in all evolutionary game-theoretical models. We have demonstrated for the first time that gestation may be an important parameter in the theoretical analysis of fair and cooperative behavior.

We found that these “gestational” constraints interacted with expected payoffs and foraging success rates to generate differential selection intensities for fairness. Selection was most intense for fairness when the reproductive delay *d* multiplied by the expected payoff on each simulation step equaled the expected resources needed to reach the reproductive threshold in *d* steps (i.e., *d* × *pR* ≅ 50 for parental investment, *P* = 0.5). When selection was most intense, juvenile social play mortality rates of 10% could still support the stable evolution of Fp. However, even when selection was not at peak intensities, Fp still evolved with approximately 2% juvenile mortality especially when the probability of obtaining resources is low but the reward was relatively high (see **Figures 3, 6**).

These conclusions also held for other values of parental investment. For parental investment of *P* = 0.7, an adult agent on average needs to accumulate at least 70 units of resources to reproduce again. For example, in simulations with an expected payoff of *pR* = 3.5, the most intense selection for fairness occurred for reproductive delays, *d*, ranging from 20 to 22, which would be expected to yield 70 to 77 units of resource. For parental investment of *P* = 0.3, an adult agent on average needs to accumulation only about 30 units of a resources to reproduce again. We found that for an expected payoff of *pR* = 3.5, the most intense selection for fairness occurred for reproductive delays, *d*, of 9, which would be expected to yield 31.5 units of resource. This suggests that there may be a previously unrecognized theoretical relationship among the evolution of fairness and cooperation in a social system, parental investment in offspring, and the minimum delay in the production of offspring. Further research will be required to more fully elucidate these relationships and their importance.

The evolution of Fp varies greatly with reproductive delay and social play mortality rates. Fp may only evolve when play mortality is relatively low, variance in payoffs is relatively high, or reproductive delays and expected payoffs are optimal for the evolution of Fp. When expected payoffs from foraging are relatively low, gestational periods that optimally support Fp are also relatively long with corresponding longer juvenile periods. Could longer periods of development facilitate acquiring more sophisticated or refined social skills? Interestingly, the experience of social play in the juvenile period appears to improve sexual performance and reproductive success in adulthood (e.g., Nunes, 2014; Ahloy Dallaire and Mason, 2017). In part, this may arise from the effects of play experience on the skills influencing social competency noted above (Marks et al., 2017). Moreover, species with more complex social play tend to have a more protracted juvenile phase (Pellis and Iwaniuk, 2000; Diamond and Bond, 2003). If so, the reproductive delays arising from the present model may reflect the longer juvenile period needed for play to train social skills. Indeed, Groos famously claimed that the purpose of youth was so they could learn or practice through play the skills they would need as adults. Although the reproductive delay is characterized as gestation in our model, it could also include postnatal parental care and investment as well.

The selection processes that emerged in these simulations were multilevel but not group selection in the classical sense. In classical group selection, a phenotype evolves because groups with individuals that possess phenotype *X* out reproduce groups without *X* leading to a proliferation of groups with *X*. In these simulations, group structure was not essential, only the social behavior of pooling resources and then dividing the pooled resources with other fair agents was essential. Similar results to those presented here can be obtained with a single large population of agents that pool resources with other fair agents in a population. Those agents that pool resources out reproduce those that do not even though all agents have the same long-term expected payoffs. Thus, selection, in these simulations, emerged from the social interactions of agents and occurred at both the individual (selfish agents) and social levels (fair agents that pool resources).

As noted in the Introduction, when animals engage in social play, they may deviate substantially from equity. In this model we assumed all have the same rate of foraging success, which justified our assumption of the even distribution of pooled resources among fair agents participating in the pool. In real-world contexts, animals have different foraging success rates and other individual differences, which may raise questions about the generalizability of this model and its results to more realistic contexts. We believe that this model and its results are likely generalizable because the benefit of equitability in resource distribution is the more efficient flow of resources into offspring under constraints. In more realistic models in which individual differences in foraging success are included, division of resources may be based on ability or contribution, but as long as, some portion of the distribution is based on equitability, there will be more efficient flow of resources into offspring than if there is no equitability at all. Thus, any strategies that tend toward equitability and thus tend to reduce inter-individual variation in resources should be selected for at the social level. This is what Schank et al. (2015) found in their evolutionary model of the dictator game. Equitability evolved among agents even though equitability did not evolve to even splits of resources. Future models could more fully investigate these complexities by introducing individual differences in individual foraging success or personality differences into fairness contexts. Empirically, we need a deeper and more precise understanding of how adult skills are acquired by engaging in social play as juveniles. For example, research correlating the frequency of self-handicapping or role-reversal behaviors in juvenile play and adult behaviors such as tolerance.

If the evolution of fairness and Fp often deviates from even-split equitability, how common is Fp in species that engage in social play? In no case that we know about, is play sustainable if play is completely inequitable (e.g., no role-reversals or no self-handicapping). This means that there may be variation in what particular pairs of play mates agree to be equitable, but whatever that level may be, it affords ample opportunity to train social skills. Although rare, play fights can escalate to serious fighting (Fagen, 1981) and this typically occurs when one of the partners fails to follow the species-typical rules that ensure that these contests remain reciprocal (Pellis and Pellis, 1998).

The proximate mechanisms regulating Fp and the acquisition of adult social skills are beyond the scope of this paper, but empirical progress has been made. During social play, any given event may lead to loss of bodily control and some pain, but playing animals have to decide whether that arose as a one off due to excessive exuberance by the partner or due to a systematic rule breaking. Such a decision requires that play partners, monitor the actions of the partner (attention), keep track of wins and losses in successive play bouts (short-term memory), do not overreact to minor transgressions (emotional regulation), and then when confronted with a major transgression take appropriate action—forgive the partner, terminate the play bout or escalate to serious aggression (decision making). In this way, playing with peers engages the executive functions of the frontal areas of the brain and there is now growing evidence that such play in the juvenile period facilitates neural development and the refinement of these skills, resulting in more socially skilled adults (e.g., Bell et al., 2010; Baarendse et al., 2013; Burleson et al., 2016; Schneider et al., 2016a,b). Moreover, it is not simply the performance of combat-like actions during play that is critical, but the modulatory adjustments needed to ensure that play fights remain reciprocal (Schneider et al., 2016a; Pellis et al., 2017). Future models could focus on more realistic learning contexts when agents engage in play fights (see Bell et al., 2015) and so identify how they may learn the rules that maintain reciprocal play.

The evolution of play and social play is likely more complicated than just whether it facilitates the acquisition of adult social skills such as cooperation (Durand and Schank, 2015) or fairness. Another recent agent-based model for the origins of play (Auerbach et al., 2015) is worth comparing to the current one as it has both important similarities and differences, as well as differing results (Auerbach et al., 2015). In this model asexually reproducing agents could engage in foraging, resting, and playing or reproducing when a certain level of energy is acquired. It differed from the current model in being a two loci model with one being an on-off play trait and the other a quantitative trait of how often the agent plays. A mutation turns play on or off. The results of various simulations showed that under conditions of ample resources in the environment, play with no fitness benefits can evolve and be maintained indefinitely, whereas play with benefits becomes both more common and more variable and thus prone to extinction. Unlike in the present model, population size was not held constant in Auerbach et al. (2015) model, but was limited by available resources. Play, being energetically costly, led to more exploitation of resources in the environment and in a resource limited environment resulted, in a counterintuitive way, to lower survival by non-playing agents. Future models could investigate the potential benefits of acquiring cooperative or fairness skills in limited resource environments where the quantity of play affects resource demand. Such models would allow us to test predictions about the environmental circumstances that favor or do not favor the evolution of Fp.

In the present model we did not consider scenarios in which agents cheat as adults. Learning how to deal with cheats also could be an important function of social play in the context of fairness. Through social play, individuals can learn who plays fairly and who does not, avoiding those that do not. Individuals could also learn how to punish cheats and thereby reduce the fitness of cheats at some cost to themselves. Future models could investigate the social-play acquisition of strategies for dealing with cheats such as punishment in public goods games (Fehr and Gächter, 2000).

Our model shows that juvenile Fp could evolve in contexts in which large packages of resources are relatively rare. In human hunter-gatherer societies, there has long been considerable evidence that relatively rare large-resource package size (e.g., meat from a large mammal) is associated with food sharing (Kaplan et al., 2000). Indeed, chimpanzees, who acquire meat much less often than human hunter-gatherers, are more likely to share meat (which usually comes in relatively larger quantities) than any other food resource they acquire (Kaplan et al., 2000). These results strongly suggest that our investigation of low-frequency large-package size resources is consistent with human evolution.

Our model may also have broad application for understanding social behavior beyond what are traditionally considered social species. Elbroch et al. (2017) reported that a solitary carnivore, the puma, often shares kills with other pumas. Puma kills are relatively rare but often involve prey several times the mass of a puma (Elbroch et al., 2017). This fits the scenarios we modeled in which resources that are large but rare generate considerable variance among individuals, in this case even being applicable to solitary pumas. According to our model, such conditions are ideal for pooling resources. Puma litters are typically 2 to 3 cubs (Logan and Sweanor, 2001), which would allow ample opportunity for littermates to learn to behave fairly via rough and tumble play. Although only a working hypothesis at this point, our model suggests that tolerance of other pumas at a kill site could be related to the degree of social play within a litter and selected because sharing large kills is most favored when resource variance is high.

Another example that could test the limits of our model occurs with Komodo dragons. Occasionally, they can bring down deer or even water buffalo by themselves. Such kills attract other Komodos, which sometimes peaceably join in the consumption of the kill or sometimes hierarchical disputes arise but sharing of kills benefit the local population (Auffenberg, 1981). Not much is known about juvenile social play in Komodos, but they do engage in playful interactions with keepers in captivity and engage in extensive play with objects in captivity becoming more solitary as they age (Burghardt et al., 2002). This suggests that it may be worthwhile to empirically investigate to what extent if any juvenile Komodos engage in social play, and if so, investigate whether aspects of their social play correlate with increased tolerance of others at their kill sites.

We are now beginning to develop a more precise and quantitative theoretical understanding of the evolution of social play and more generally, play. Play can evolve to facilitate adult cooperation even when social play among juveniles is costly (Durand and Schank, 2015). Here we have shown that Fp can evolve to facilitate fairness in adults and this may provide further theoretical insights for empirical studies investigating Fp in different species. However, Auerbach et al. (2015) model suggest that understanding the evolution of play is not as simple as just weighing adult benefits against social play costs. Play may evolve without any functional benefit under conditions of abundant resources. Although, once present, play may readily be co-opted for novel functional benefits (Pellis et al., 2015).

Future models could investigate richer and more detailed social play contexts in which juveniles not only learn to behave fairly and cooperate as adults, but also learn through their play interactions strategies for dealing with cheats and unfair individuals. Models could also investigate individual differences such as foraging abilities in adults or age difference interactions in juvenile play. Models such as Auerbach et al. (2015) can be extended to further investigate the conditions under which play can evolve. Differences in personalities or behavioral syndromes both in juvenile play and adult behavior also may be important to include in future models (e.g., see Sih et al., 2004). In our view, developing a theory of the evolution of play involves developing a family of related and increasingly testable models. Our model takes us another step toward this long-term goal.

## Author Contributions

JS developed the model, helped conceptualize the project, and wrote the first draft. GB and SP helped conceptualize the project and helped write and edit the manuscript.

## Conflict of Interest Statement

The authors declare that the research was conducted in the absence of any commercial or financial relationships that could be construed as a potential conflict of interest.

## Footnotes

1 www.scientificlinux.org
